# Hydrothermal Treatment Modifies the Chemical Profile of Coffee Pulp (*Coffea arabica*) Extracts and Improves Their Antibacterial Activity against *Xanthomonas* and *Ralstonia*

**DOI:** 10.1021/acsomega.6c07937

**Published:** 2026-10-23

**Authors:** Eddy Jirón-García, Karina Rodríguez-Mora, Lidieth Uribe, Cesar Bernal-Samaniego

**Affiliations:** † 27915 Sede Regional Del Caribe, Universidad de Costa Rica, Limón, Limón 70101, Costa Rica; ‡ Instituto de Investigaciones En Ingeniería (INII), Universidad de Costa Rica, San Pedro, San José 2060, Costa Rica; § Centro de Investigaciones Agronómicas, Universidad de Costa Rica, San Pedro, San José 2060, Costa Rica

## Abstract

Coffee pulp is an abundant agro-industrial residue whose bioactive compounds could contribute to integrated valorization strategies. This study evaluated the *in vitro* antibacterial activity and chemical profiles of methanolic extracts from untreated and hydrothermally treated coffee pulp against bacteria of the genera *Xanthomonas* and *Ralstonia* associated with crops in Costa Rica. Coffee pulp was treated at 170 °C for 1 or 3 h, and the resulting solid residues were subjected to ultrasound-assisted extraction with 50% (v/v) aqueous methanol. After concentration, the extract obtained after 1 h of hydrothermal treatment showed the highest total polyphenol content (2,100 ± 300 mg of gallic acid equivalents per gram of dry biomass), whereas the extract obtained after 3 h exhibited the greatest inhibition against *Xanthomonas* (68 ± 5%) and *Ralstonia* (90 ± 7%), compared with 57 ± 7% and 58 ± 10%, respectively, for untreated coffee pulp. Chemical profiling by high-performance liquid chromatography coupled to high-resolution mass spectrometry indicated glycosylated flavonoids and triterpenes in untreated pulp extract, whereas the hydrothermally treated samples contained phenolic aldehydes, indoles, oxylipins, and amphiphilic lipids, which may be associated with their greater inhibitory activity. These findings show that hydrothermal treatment modified the chemical profile of coffee-pulp extracts and increased their inhibitory activity against *Xanthomonas* and *Ralstonia*, highlighting its potential as a pretreatment for the valorization of this agro-industrial residue.

## Graphical abstract

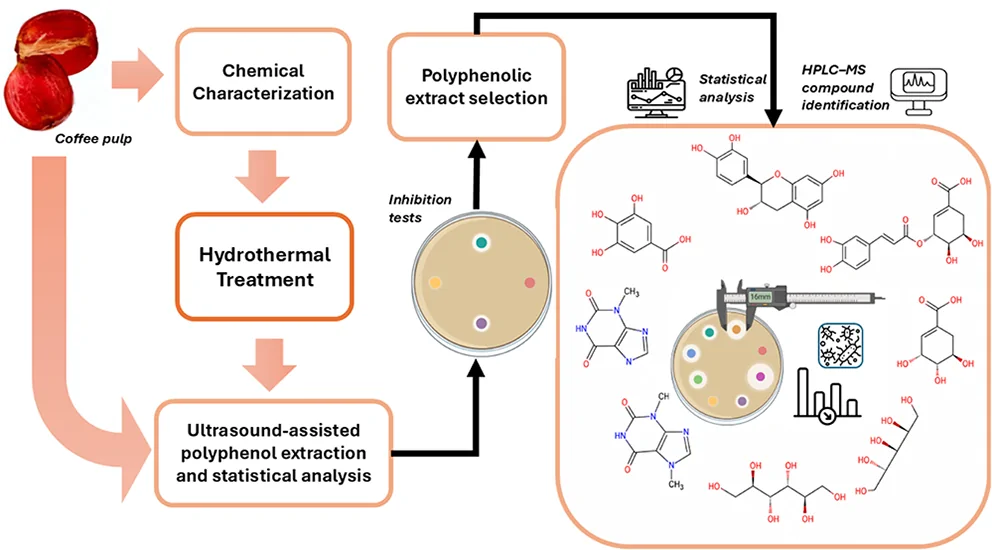

## Introduction

The global population is increasing at an unprecedented rate, which creates an urgent need to ensure food security. It is estimated that by 2050, global food production must be sufficient to sustain approximately 9.7 billion people. Consequently, considerable efforts have been directed toward achieving intensive agricultural production, which has led to a continuous increase in the level of pesticide use. Over the past decade, global pesticide use in agriculture has risen by approximately 13%.^1^ Although this intensification has contributed to maintaining agricultural productivity in line with population growth, it has also resulted in significant adverse effects on human health, soil quality, water resources, and biodiversity.^1–3^

Coffee is one of the most widely traded agricultural commodities worldwide. During the 2022–2023 period, global coffee production reached 165.5 million 60 kg bags, of which 56.7% corresponded to the *Arabica* variety.^4^ Coffee cultivation has a significant economic and social impact across more than 50 producing countries, including those in Central and South America, Mexico, and East Africa. This sector generates approximately 40.68 million tons of biomass annually, representing both a waste management challenge and an opportunity for the implementation of circular economy strategies through the valorization of byproducts such as husks, pulp, and other processing residues.^4^

Given that Costa Rica is an agro-exporting country, coffee is positioned as one of the country’s main export products, accounting for 5.9% of total agricultural exports.^5^ Although it was surpassed by pineapple and banana during the 2022–2023 period, according to the latest Agricultural Report, coffee remained a strategic crop due to its economic impact, its territorial relevance in coffee-growing regions, and its traditional contribution to exports.^5^

Coffee pulp is a major byproduct generated during coffee processing and is rich in organic compounds. It contains approximately 21–32% carbohydrates, 7.5–15% proteins, and 2–7% lipids on a dry weight basis.^6^ In addition, coffee pulp is an important source of bioactive compounds, including tannins (2–9%), carbohydrates (21–50%), caffeine (1–2%), chlorogenic acids (2–3%), and caffeic acid (2%) (dry weight basis).^7^ The abundance of these valuable biomolecules has attracted increasing interest in the valorization of coffee pulp as a sustainable feedstock to produce value-added products in food, pharmaceutical, agricultural, and biotechnological applications.^8^ The Costa Rican coffee industry generates approximately 250,802 t of coffee pulp each year.^9^ In the absence of appropriate management strategies, this agro-industrial residue constitutes a significant environmental challenge due to its high organic load, which can lead to soil and water pollution while adversely affecting surrounding ecosystems and nearby communities.^9^

Polyphenolic compounds obtained from coffee pulp extracts represent an environmentally friendly and accessible option for pest control in coffee-producing countries. This alternative is characterized by low environmental impact, high biodegradability, and a low risk of toxicity to nontarget organisms. However, their application in the control of phytopathogenic bacteria requires further investigation.^10^

The bioactive potential of polyphenolic extracts positions them as a promising alternative to antibiotics commonly used in Costa Rican agriculture, such as oxytetracycline, oxytetracycline–streptomycin mixtures, and the aminoglycoside kasugamycin.^11^ In Costa Rica, several phytopathogenic bacterial genera affecting economically important crops have been reported, including *Burkholderia*,^12^ *Enterobacter*,^13^ *Erwinia* associated with mango,^14^ banana,^15^ and pineapple,^16^ *Pectobacterium*,^17^ *Xylella*, which affects coffee, avocado, ornamental plants, and citrus trees,^18^ *Xanthomonas*,^19^ and *Ralstonia* species associated with bacterial wilt in tomato crops.^20,21^

This work focuses on the extraction of polyphenols from coffee pulp using ultrasound-assisted extraction (UAE) and the evaluation of the antibacterial activity of the resulting polyphenolic extracts and their fractions against two selected phytopathogenic bacteria affecting economically important crops in Costa Rica. This approach is proposed as a sustainable alternative to conventional chemical control methods. The selected bacterial pathogens were *Xanthomonas* spp. and *R. solanacearum*.

Bacteria belonging to the genus *Xanthomonas* infect a wide range of plant tissues including leaves, stems, pods, and seeds. Seed-borne transmission represents a major challenge for disease management because it facilitates pathogen dissemination and reduces the effectiveness of conventional control measures. Moreover, numerous pathovars and physiological races have been identified within the genus, each exhibiting distinct host specificities toward different plant genotypes. This high degree of pathogenic variability complicates the development of resistant crop varieties and highlights the need for alternative disease management strategies.^22^

The *R. solanacearum* species complex (RSSC), on the other hand, causes diseases of high phytosanitary relevance due to its negative impact on agricultural yields. In particular, the RALSA-MA isolate, classified as *R. solanacearum* (phylotype II), is able to persist in soil, colonize vascular tissues, and cause significant economic losses in crops such as tomato, potato, and other solanaceous plants.^23,24^

## Materials and Methods

### Sample Preparation

Fresh coffee pulp (*Coffea arabica*) generated during the processing of ripe coffee cherries was supplied by the Coopetarrazú Coffee Mill, located in Bajo San Juan, San Marcos, Costa Rica. The coffee pulp was dried in a TABLEMAZ tray convection oven at 103 °C for 36 h. The dried material was ground using a Retsch SM300 knife mill operating at 1,300 rpm and sieved to obtain a particle size of 1 mm.

### Chemical Analysis

The chemical composition of the coffee pulp was determined following TAPPI Standard T280 pm-99 and the National Renewable Energy Laboratory (NREL) analytical procedures NREL/TP-510-42620 and NREL/TP-510-42618. These methods were used to quantify the contents of extractives, cellulose, hemicellulose, lignin, and ash in the coffee pulp samples.^25^

### Experimental Workflow

The experimental workflow used for ultrasound-assisted extraction method selection, sample generation, preliminary screening, antibacterial evaluation, and sequential fractionation is summarized in Figure 1.

**Figure 1:**
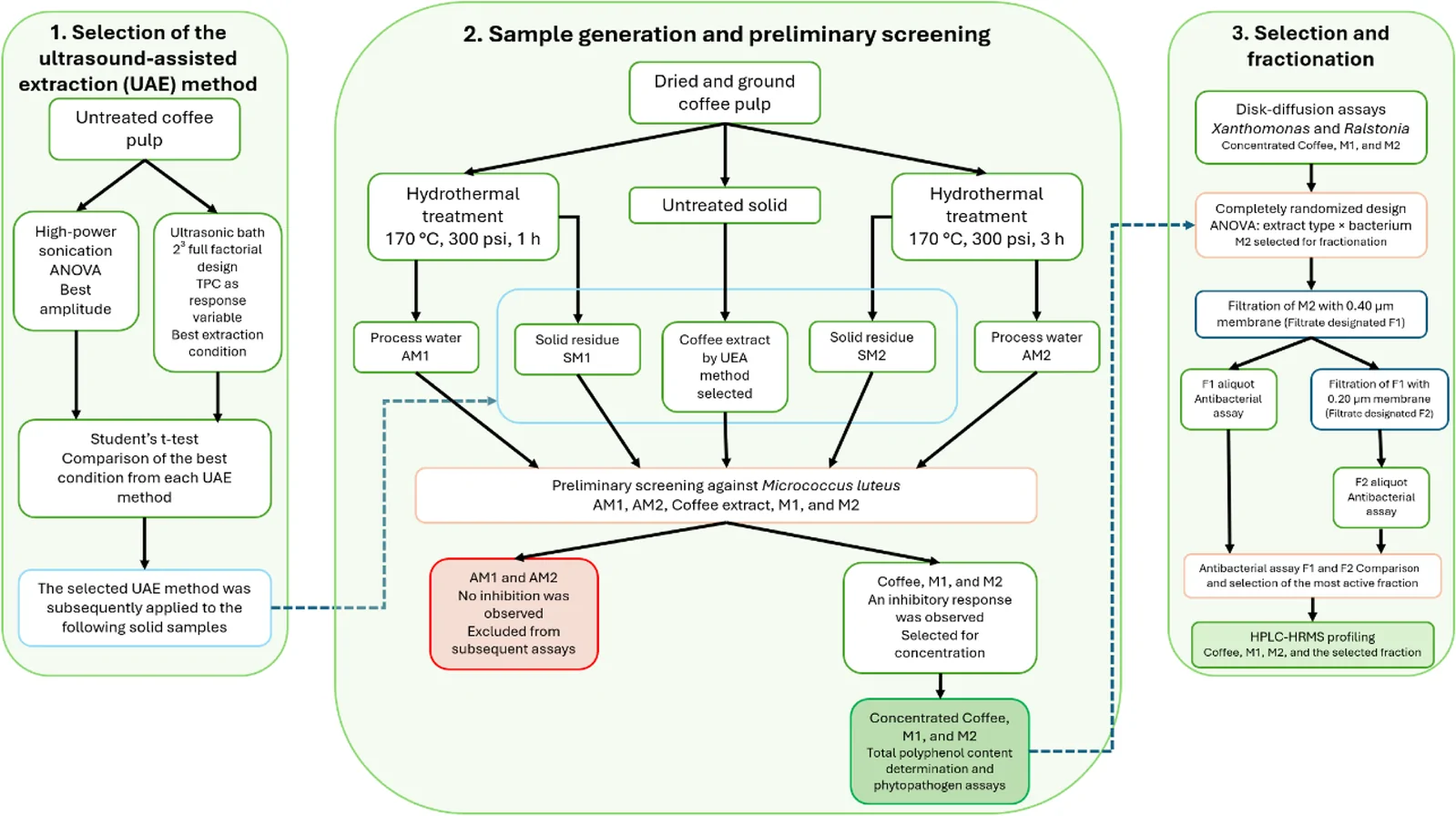
Experimental workflow for the selection of the ultrasound-assisted extraction method and the generation, evaluation, and fractionation of coffee-pulp extracts.

### Selection of the Ultrasound-Assisted Extraction Method

#### High-Power Sonication

To select the ultrasound-assisted extraction (UAE) method, high-power sonication and ultrasonic bath extraction were evaluated using untreated dried and ground coffee pulp (Figure 1). High-power sonication was performed for 3 min at amplitudes of 5, 10, and 15%, using a biomass-to-solvent ratio of 1 g:25 mL and 50% (v/v) aqueous methanol as the extraction solvent. An ice bath was used to prevent overheating during the sonication. The short sonication time was selected to minimize solvent evaporation. The total polyphenol content was measured in triplicate, and the effect of amplitude was analyzed by analysis of variance (ANOVA) at a 95% confidence level. The amplitude yielding the highest total polyphenol content was selected as the best conditions for high-power sonication

#### Ultrasonic Bath Extraction

For ultrasonic bath extraction, 50% (v/v) aqueous methanol was used as the extraction solvent.^26^ A full factorial design was used to evaluate extraction time, temperature, and biomass-to-solvent ratio, with total polyphenol content as the response variable (Table 1). The biomass-to-solvent ratio of 1:12 g:mL was the lowest feasible ratio because lower solvent volumes did not completely wet the porous material. Each experimental condition was evaluated in triplicate. The best condition obtained for ultrasonic bath extraction was selected based on the total polyphenol content.

**Table 1:** Experimental Design of the 2^3^ Full Factorial Design Used for Ultrasound-Assisted Extraction in an Ultrasonic Bath

		Levels	
Factors		Low	High
(A)	Time (min)	20	40
(B)	Biomass-to-solvent ratio (g:mL)	1:12	1:25
(C)	Temperature (°C)	25	40

#### Comparison and Selection of the UAE Method

The best conditions obtained for high-power sonication and ultrasonic-bath extraction were then compared using Student’s *t*-test at a 95% confidence level. Statistical analyses were performed using RStudio version 2024.12.0 + 467. The UAE principle yielding the highest total polyphenol content condition was selected for subsequent experiments.

#### Determination of Total Polyphenol Content

The total polyphenol content (TPC) was determined using a modified Folin–Ciocalteu method.^27^ Briefly, 100 μL of sample or blank solution was transferred into a 2 mL reaction tube, followed by the addition of 200 μL of diluted Folin–Ciocalteu reagent (1:10, v/v, in distilled water). The mixture was vortexed, and 800 μL of 700 mM Na_2_CO_3_ solution was subsequently added. The reaction mixture was incubated at room temperature for 2 h, after which the absorbance was measured at 679.5 nm using a UV–vis spectrophotometer. The total polyphenol concentration was quantified using a gallic acid calibration curve prepared with standard solutions ranging from 50 to 110 mg L^–1^. The results were expressed as milligrams of gallic acid equivalents per gram of dry biomass (mg GAE g^–1^ DW).

### Sample Generation and Preliminary Screening

#### Hydrothermal Treatment and Sample Generation

Dried and ground coffee pulp was subjected to hydrothermal treatment^27^ as summarized in Figure 1. A biomass-to-water ratio of 100 g:2 L (pulp:water) was used, and the mixture was treated at 170 °C and 300 psi for 1 or 3 h. After the 1 h treatment, the liquid fraction (process water) and solid residue were designated AM1 and SM1, respectively. After the 3 h treatment, the corresponding liquid fraction and solid residue were designated AM2 and SM2, respectively. Total polyphenol content was quantified in the process waters.

The selected UAE method was subsequently applied to untreated coffee pulp, SM1, and SM2, producing coffee, M1, and M2 extracts, respectively. Thus, five samples were generated for preliminary screening: the process waters AM1 and AM2 and the UAE extracts coffee, M1, and M2.

#### Preliminary Screening and Extract Concentration

All samples were initially evaluated for antibacterial activity by using *Micrococcus luteus* as an indicator microorganism. AM1 and AM2 showed no inhibitory activity and were excluded from the subsequent assays. Coffee, M1, and M2 showed limited inhibitory activity under the initial extraction conditions and were, therefore, selected for concentration. The concentration procedure is summarized in Figure S1. The biomass-to-solvent ratio during UAE was reduced from 1:12 to 1:3.6 g:mL, followed by methanol removal and freeze-drying. The total polyphenol content was subsequently determined for the concentrated coffee, M1, and M2 extracts. These extracts were then evaluated against the phytopathogenic bacteria *Xanthomonas* sp. and *R. solanacearum*, as described below.

### Selection and Fractionation

#### Antibacterial Assays

The concentrated coffee, M1, and M2 extracts were evaluated against *Xanthomonas* sp. and *Ralstonia solanacearum*, provided by the Centro de Investigación en Biología Celular y Molecular (CIBCM), as summarized in Figure 1. Antibacterial activity was assessed by the disk-diffusion method. Bacterial suspensions adjusted to the 0.5 McFarland standard, equivalent to CFU/mL, were inoculated onto Mueller–Hinton agar. Sterile 6 mm-diameter disks loaded with 10 μL of each test extract were placed on the inoculated agar, and plates were incubated at 32 °C for 48–72 h in a BTM Friocell incubator.

Gentamicin at 0.1 mg/mL and the extraction solvent were used as the positive and negative controls, respectively. The inhibition percentage was calculated according to eq 1, adapted from a previously reported method^28^ where *I* represents the inhibition percentage (%) and *D_I_, D_C_, and D_N_* represent the inhibition diameters of the extract, positive control, and negative control, respectively.

1 $$I=\frac{D_{I}-D_{N}}{D_{C}-D_{N}}\cdot 100$$

A completely randomized design with five replicates was used to evaluate the effects of extract type, bacterial species, and their interaction on the inhibition percentage.

### Sample Fractionation

Based on its overall inhibitory response, M2 was selected for fractionation. M2 was first filtered through a 0.40 μm cellulose acetate membrane, and the filtrate was designated F1. An aliquot of F1 was evaluated for antibacterial activity, whereas the remaining F1 was subsequently filtered through a 0.20 μm membrane to obtain F2. Thus, the sequential microfiltration procedure generated two filtrate fractions, F1 and F2. Their antibacterial activities were compared statistically to select the fraction with the highest activity for subsequent UHPLC-HRMS analysis.

### UHPLC-HRMS Characterization of the Extracts

The concentrated coffee, M1, M2, and F2 samples were characterized by UHPLC-HRMS. Analyses were performed at the Centro de Investigaciones en Productos Naturales (CIPRONA), Universidad de Costa Rica. Samples were filtered through 0.22 μm membrane filters, dried, and reconstituted in 80% (v/v) methanol in water at 5 mg/mL.

Chromatographic separation was performed using a Vanquish UHPLC system coupled to an Orbitrap Exploris 120 high-resolution mass spectrometer equipped with an HESI-II source. A Waters HSS T3 C18 column (150 × 2.1 mm, 1.8 μm) was maintained at 45 °C. Water containing 0.1% formic acid (A) and acetonitrile containing 0.1% formic acid (B) were used as mobile phases at 400 μL/min, with an injection volume of 1 μL. The gradient was 2% B from 0 to 2 min, 40% B at 12 min, and 98% B from 18 to 21 min, followed by return to the initial conditions and re-equilibration until 26 min.

Data-dependent acquisition was conducted in positive and negative ionization modes over an *m*/*z* range of 100–1500 at a resolving power of 30,000 at *m*/*z* 200. The spray voltage, vaporizer temperature, and capillary temperature were 3.5 kV, 300 °C, and 320 °C, respectively. Sheath, auxiliary, and sweep gas settings were 45, 15, and 5 arbitrary units, respectively, and the S-lens RF level was set to 70. Data were acquired using Xcalibur version 4.5 and processed for putative compound annotation using GNPS2 and SIRIUS.

## Results and Discussion

The chemical characterization presented in Table 2 confirmed the lignocellulosic nature of coffee pulp. These values are consistent with those reported for coffee pulp in previous studies.^29–31^ The lignin content is relevant to hydrothermal treatment because it provides an aromatic fraction that can be modified during processing and can contribute to the recovery of phenolic compounds. Together with the cellulose and hemicellulose contents, this composition supports the use of coffee pulp for sequential hydrothermal treatment and UAE.^32,33^

**Table 2:** Chemical Characterization of Coffee Pulp

Parameter	Mean ± standard deviation (% w/w)
Extractives	1.4 ± 0.4
Cellulose	21.4 ± 1.8
Hemicellulose	37.6 ± 2.4
Lignin	16.6 ± 1.6
Ash	2.2 ± 0.3

The compositional profile also identifies potential routes for valorizing residual biomass after polyphenol recovery. The high hemicellulose and cellulose contents indicate potential for subsequent biological conversion processes, whereas the wet residue generated after hydrothermal treatment could be directed to anaerobic digestion, which has been reported as a viable valorization strategy.^34^ Alternatively, if the residual biomass is directed toward material applications, delignification would be required to separate cellulose for its subsequent conversion into high-value materials such as nanocellulose.^35^ Pyrolysis and gasification represent additional thermochemical alternatives; however, their application would require moisture reduction before processing, adding an energy demand that should be considered in a future technoeconomic evaluation.^36^

### Selection of the Ultrasound-Assisted Extraction Method

The ultrasonic frequency and exposure time play a crucial role, as the extraction mechanism relies on the generation, growth, and collapse of microbubbles through acoustic cavitation. This phenomenon disrupts the plant cell structure, enhances mass transfer, and facilitates the release of intracellular polyphenolic compounds, thereby improving extraction efficiency.^37^ Figure S2 shows a comparison between both ultrasound-assisted extraction methods .

The total polyphenol content decreased as the sonication amplitude increased. Statistical analysis revealed no significant differences between amplitudes of 5% and 10%, whereas significant differences were observed between 5% and 15% and between 10% and 15%. This behavior may be attributed to the effect of high ultrasonic amplitudes on the cavitation process. Excessive amplitude can reduce the efficiency of cavitation by promoting bubble coalescence and unstable bubble collapse, thereby limiting the mass transfer. In addition, high-intensity sonication favors the formation of reactive oxygen species, particularly hydroxyl radicals, which can oxidize and degrade the extracted polyphenolic compounds, ultimately reducing the total polyphenol yield;^38,39^ as a result, lower total polyphenol contents were obtained. Furthermore, when using the high-power ultrasonic probe, higher sonication amplitudes require shorter extraction times because the greater acoustic energy delivered per unit volume leads to a rapid increase in the solvent temperature. This temperature increase may promote the degradation of thermolabile phenolic compounds and the evaporation of the solvent.^39^

The full 2^3^ factorial design showed that the biomass-to-solvent ratio was the only factor with a significant effect on the total polyphenol content. As shown in the standardized Pareto chart (Figure S3), factor B, corresponding to the biomass:solvent ratio, had the greatest effect on the response, whereas extraction time (factor A) and temperature (factor C) were not significant within the evaluated ranges. The lower ratio, 1 g:12 mL, resulted in a higher total polyphenol content than the 1 g:25 mL ratio.

The selected experimental ranges were appropriate for preserving polyphenolic compounds during UAE. Temperatures above 40 °C and prolonged sonication have been associated with the degradation of phenolic compounds,^39,40^ whereas the biomass-to-solvent ratio is recognized as a relevant variable affecting polyphenol extraction from plant matrices.^41^ The use of 50% (v/v) aqueous methanol was also consistent with previous studies reporting the suitability of aqueous organic-solvent mixtures for the recovery of phenolic compounds^42–44^ as higher solvent polarity increases the solubility of these compounds.^45^

The factorial design enabled the evaluation of the combined effects of the extraction variables, as illustrated by the interaction plots in Figure S3. Rather than performing a comprehensive optimization, the design was used to identify the treatment combination providing the highest total polyphenol content within the evaluated operating range. This response was obtained at 40 min, a biomass-to-solvent ratio of 1 g:12 mL, and 40 °C. The ultrasonic bath under these conditions was subsequently compared with the best condition obtained by high-power sonication. The Student’s *t-test* showed a significant difference between the UAE methods (t = −11.81, *p* < 0.0001), and the ultrasonic bath was selected for the subsequent experiments.

The total polyphenol content of the coffee pulp was determined to be 220 ± 9 mg GAE g^–1^ DW, a value within the range previously reported for coffee-pulp extracts obtained using assisted-extraction methods,^40^ confirming that this agro-industrial byproduct is a rich source of bioactive compounds and a promising feedstock for polyphenol extraction.

Although the total polyphenol contents reported for coffee pulp vary substantially across studies, these differences can largely be attributed to the condition of the biomass (fresh, dried, or processed), the extraction method (conventional maceration, ultrasound-assisted extraction, microwave-assisted extraction, or their combination), and the extraction solvent used, all of which significantly influence the recovery of phenolic compounds.^40,46,47^ In this context, the combination of thermal pretreatment to facilitate the release of polyphenols bound to the lignocellulosic matrix with subsequent ultrasound-assisted extraction may constitute an effective approach to improve the extraction yield.^47^ Nevertheless, the impact of this strategy on the antibacterial activity of the extracts should be experimentally evaluated to ensure that the inhibitory potential is maintained or is enhanced.

### Sample Generation and Preliminary Screening

Hydrothermal treatment can disrupt the lignocellulosic matrix and facilitate the release of phenolic compounds associated with the biomass, although excessive thermal exposure may promote the degradation of heat-sensitive compounds. Previous studies, mainly conducted with spent coffee grounds, have reported improvements in polyphenol recovery after hydrothermal pretreatment.^27,47^ Under the conditions evaluated in this study, UAE of the solid residues obtained after 1 and 3 h of hydrothermal treatment yielded the M1 and M2 extracts, with total polyphenol contents of 220 ± 8 and 228 ± 5 mg GAE g^–1^ DW, respectively. These values were similar to that of untreated coffee pulp (220 ± 9 mg GAE g^–1^ DW), indicating that hydrothermal treatment produced only a slight increase in the total polyphenol content recovered from the solid fraction.

The process waters obtained after the 1 and 3 h treatments, designated AM1 and AM2, contained 221 ± 6 and 221 ± 9 mg GAE g^–1^ DW, respectively. The similar polyphenol contents of AM1 and AM2 indicate that extending the treatment time from 1 to 3 h did not substantially change the number of detectable compounds in the aqueous phase. This behavior is consistent with the distribution of phenolic compounds between the process water and the solid fraction during hydrothermal treatment. Previous work has shown that lignin hydrolysis can contribute to phenolic compounds in the liquid phase, while organic compounds associated with the solid surface may coexist in equilibrium with dissolved species.^27^ In this context, the lignin content reported in Table 2 is relevant because its partial degradation may facilitate the release of phenolic compounds otherwise bound or entrapped within the lignocellulosic matrix.^27^

The distribution of phenolic compounds between both fractions depends on their chemical structure, including the number and position of hydroxyl (−OH) and methoxy (−OCH_3_) groups. More soluble compounds may be transferred to the process water, where they are more susceptible to hydrolysis, oxidation, and other transformation reactions as treatment severity increases. In contrast, compounds more strongly associated with the lignocellulosic matrix may remain in the solid residue and be subsequently recovered by methanolic UAE. Thus, the solid fraction may retain phenolic compounds more strongly associated with the lignocellulosic matrix, which can subsequently be recovered during methanolic UAE.^41^

*Micrococcus luteus*, a Gram-positive bacterium commonly used in assays of this type as a model organism due to its rapid growth, low pathogenicity, good diffusion in the agar used, and response to antibiotics and natural extracts, was used.^48–50^

As observed in Figure 2, no evidence of bacterial growth inhibition was observed in the disks corresponding to the process waters referred to as AM1 and AM2 in the figure. In contrast, the extracts obtained by ultrasound-assisted extraction (UAE) showed very slight inhibitory activity, as denoted by an inhibition halo of less than 1 mm in diameter (see close-up in Figure 2). This difference may be attributed to the origins of the polyphenols present in each sample. The process waters contain degraded compounds, since the hydrothermal pretreatment used aims to break down lignin and hemicellulose to facilitate subsequent extractions,^32^ thereby generating liquids rich in simple carbohydrates and low-molecular-weight phenols.^33^ On the other hand, the extracts obtained by UAE retain polyphenolic compounds derived from the residual lignin in the biomass, which are solubilized in methanol used for the extraction.

**Figure 2:**
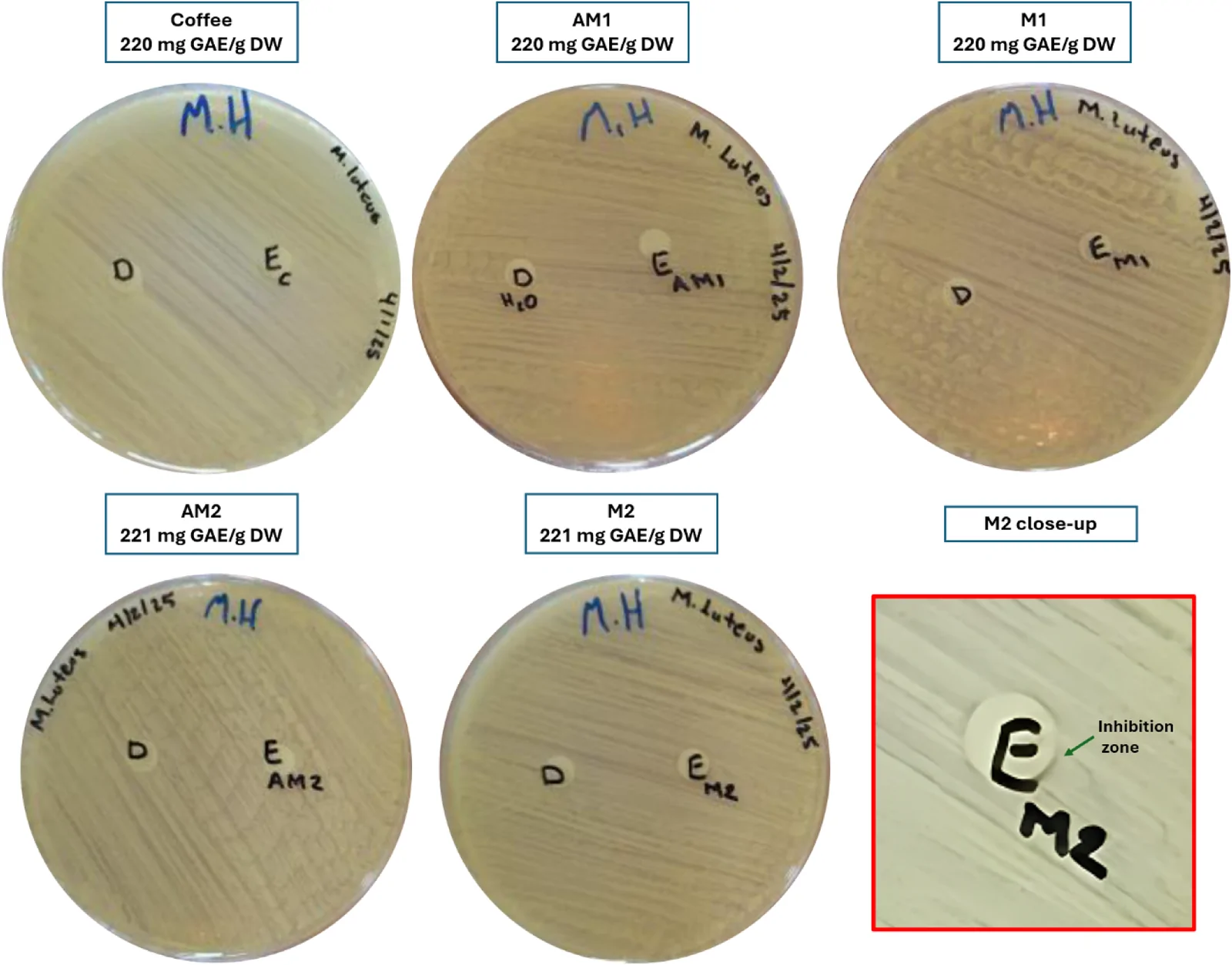
Preliminary inhibition tests using *Micrococcus luteus* as the indicator bacterium, where D corresponds to the solvent and E corresponds to the extract. Coffee: extract in 50% methanol obtained from coffee pulp; AM1 and AM2: process waters from the hydrothermal treatments of M1 (170 °C for 1 h) and M2 (170 °C for 3 h), respectively; M1 and M2: extracts in 50% methanol obtained from the solid fractions treated under the same conditions.

Figure 2 provides valuable information regarding the influence of the solvent on bacterial inhibition as a negative control. The solvent used was 50% methanol to compare the coffee, M1, and M2 extracts, while sterile water was used in the tests of the process waters. It is shown that, although methanol has certain toxicity, both environmental and to general health,^51^ the concentration used did not affect the growth of the model bacterium; therefore, if inhibition were observed in subsequent tests, it would correspond only to the influence of the extract and not to the solvent. Taking the above into account, it was decided not to use the process waters in the extract concentration stages since, despite containing polyphenols, they showed no evidence of inhibitory activity.

The concentration methods consisted of removing methanol from the prepared extracts followed by freeze-drying, since this helps achieve complete solvent reduction and preserve activity,^52^ while the other method involved reducing the volume during the UAE stage in the ultrasonic bath.^53^ The effects observed after removing methanol from the prepared and freeze-dried extracts showed results similar to those in Figure 2, despite showing an increase in concentration. This is attributed to the probable degradation of the compounds during the methanol evaporation process due to prolonged periods at a temperature below 40 °C but above 30 °C.

When the solvent volume was reduced during the UAE stage, a greater increase in the total polyphenol content was observed for each sample. In this case, the maximum increases were obtained by decreasing the volume from 12 to 3.6 mL per gram of sample. This volume is important because the hydrothermal treatment increases the porosity of the solid matrix, which provides liquid absorption properties; consequently, volumes lower than 3.6 mL were not sufficient to cover the sample. Table 3 shows the total polyphenol content of the extracts obtained by reducing the solvent-to-biomass ratio to 3.6 mL/g during ultrasound-assisted extraction. At the same time, Figure 3 shows that inhibition was effectively observed in both the indicator and phytopathogenic bacteria for sample M2.

**Table 3:** Total Polyphenols in the Extracts Obtained Using a Ratio of 3.6 mL, a Temperature of 40 °C, and a Time of 40 min in the Ultrasonic Bath

Extract	Total polyphenols ± standard deviation (mg GAE/g DW)
Coffee	1,100 ± 200
M1	2,100 ± 300
M2	1,300 ± 100

**Figure 3:**
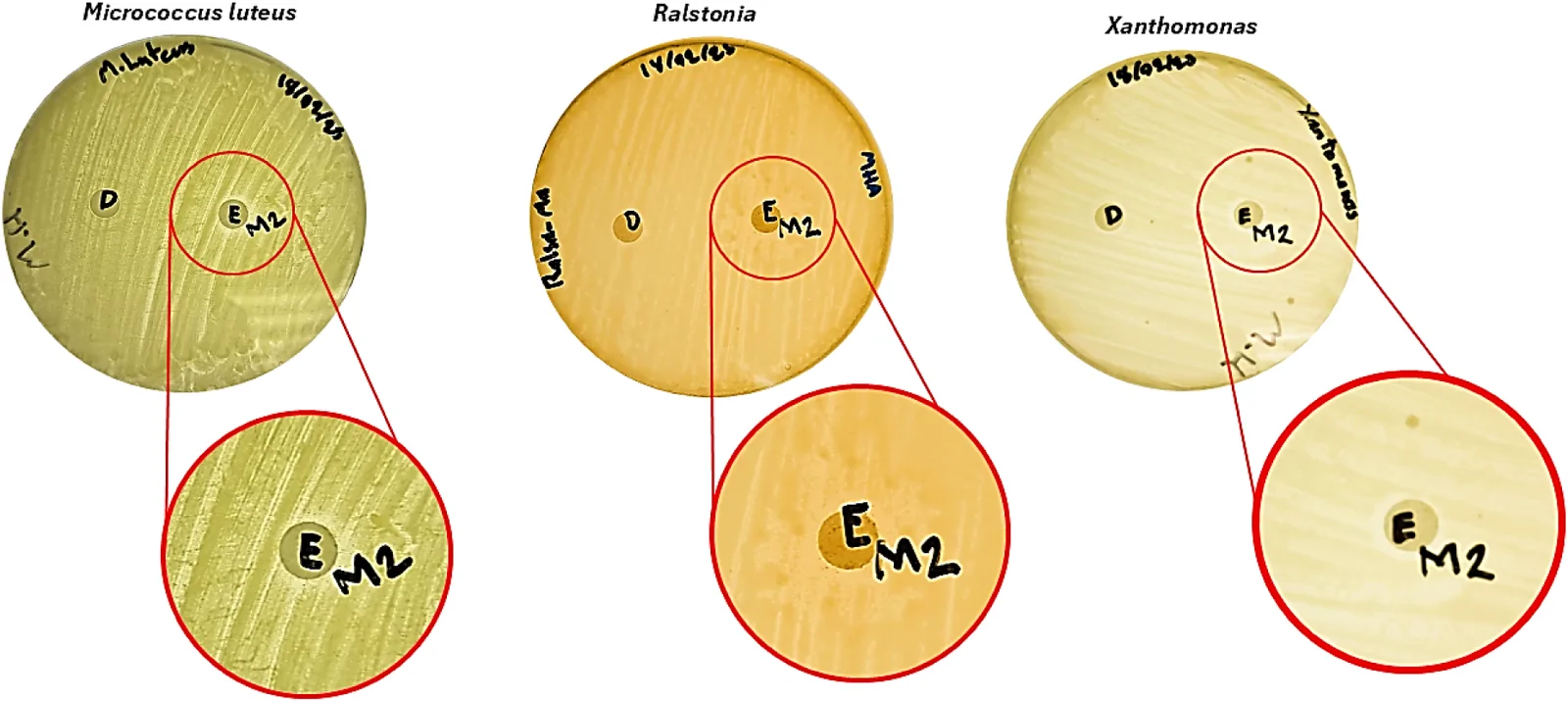
Inhibition produced by the M2 extract after concentration using a 3.6 mL/g ratio and its effect on *Micrococcus luteus* as the indicator bacterium and on *Ralstonia*and *Xanthomonas* as phytopathogenic bacteria.

### Selection and Fractionation

Figure 3 shows the inhibition of the model bacterium; however, the inhibition zone is not a circular halo but has an irregular shape. This shape results from bacterial streaking and its diffusion in the agar. Although the mechanism of action of polyphenols is complex, they exhibit antibacterial activity mainly through the alteration of key structures such as the cell wall and cell membrane, generating changes in osmotic pressure and an increase in ionic forces within the bacterium.^54^ Another possible mechanism of action is an interaction that changes the fluidity of the membrane lipid bilayer and makes it more permeable, compromising important cellular processes such as ion transport and respiration, causing the loss of essential compounds, metabolic disruption, and ultimately bacterial death.^55^

At the same time, Figure 3 shows inhibition of the phytopathogenic bacteria.^24,56^ Although both bacteria are Gram-negative and have a different cell membrane structure from Gram-positive bacteria such as *Micrococcus luteus*, inhibition was still observed. In general, the affinity and penetration of polyphenols depend on the species present in the extract. This is because Gram-negative bacteria possess an outer lipopolysaccharide membrane that makes them more resistant to the action of these compounds, compared with Gram-positive bacteria, whose peptidoglycan wall allows greater permeability.^57^

Table 4 shows the average inhibition percentages of the phytopathogenic bacteria. Likewise, Tables S1–S3 show the data from the disk-diffusion assays. The statistical analysis showed that M1 and M2 had a significantly greater effect than the coffee extract on inhibition zones. A significant interaction was observed between the type of extract and the bacterium evaluated, indicating that the effect of the extract is dependent on the microorganism. Overall, the results show that the M1 and M2 extracts differ significantly from the coffee extract, although they do not show significant differences between each other. However, the M2 extract was selected for fractionation because it showed a slight increase in the inhibition percentage.

**Table 4:** Calculated Inhibition Percentage for Each Extract against the Evaluated Phytopathogenic Bacteria

Extract	*Xanthomonas* *(%)*	*Ralstonia* *(%)*
Coffee	57 ± 7	58 ± 10
M1	64 ± 4	82 ± 17
M2	68 ± 5	90 ± 7

These results are noteworthy because copper-based compounds are used to manage *Xanthomonas*.^22,58^ As shown in Tables S1–S3, the untreated coffee-pulp, M1, and M2 extracts produced inhibition zones of 9–11, 10–11, and 10–11 mm against *Xanthomonas*, respectively. Comparing this with other authors using copper compounds, zones of 9.81–14.55 mm have been reported for copper compounds at 5000 μg/mL.^59^ Against *Ralstonia*, the corresponding values were 7–8, 9–10, and 10–12 mm, respectively, while other authors reported 6.45–12.30 mm for copper compounds at 1000–4000 ppm.^60^ Although direct chemical control of *Ralstonia* relies mainly on phytosanitary management,^61^ these literature-based comparisons indicate that all three extracts produced inhibition zones within the range reported for some copper-based treatments, with the effect of hydrothermal treatment being most evident against *Ralstonia*.

Although both bacteria are Gram-negative, *Ralstonia* and *Xanthomonas* differ in their colonization strategies and virulence-associated traits, which may influence their interactions with the complex chemical composition of the extracts.^62^ These differences may contribute to the significant extract-bacterium interaction observed, although the underlying mechanisms were not evaluated in this study.

Figure 4 shows the results of the M2 extract separation, in contrast with the other extracts. It is observed that the effectiveness depends on the bacterium. In the case of *Ralstonia*, all extracts were significantly more effective than the coffee extract, whereas no significant differences were found among the extracts for this bacterium. In the case of *Xanthomonas*, no significant differences were found among the extracts, although F2 and M2 showed a tendency for greater inhibition. On the basis of these bacterium-dependent responses, coffee, M1, M2, and F2 were selected for UHPLC-HRMS characterization.

**Figure 4:**
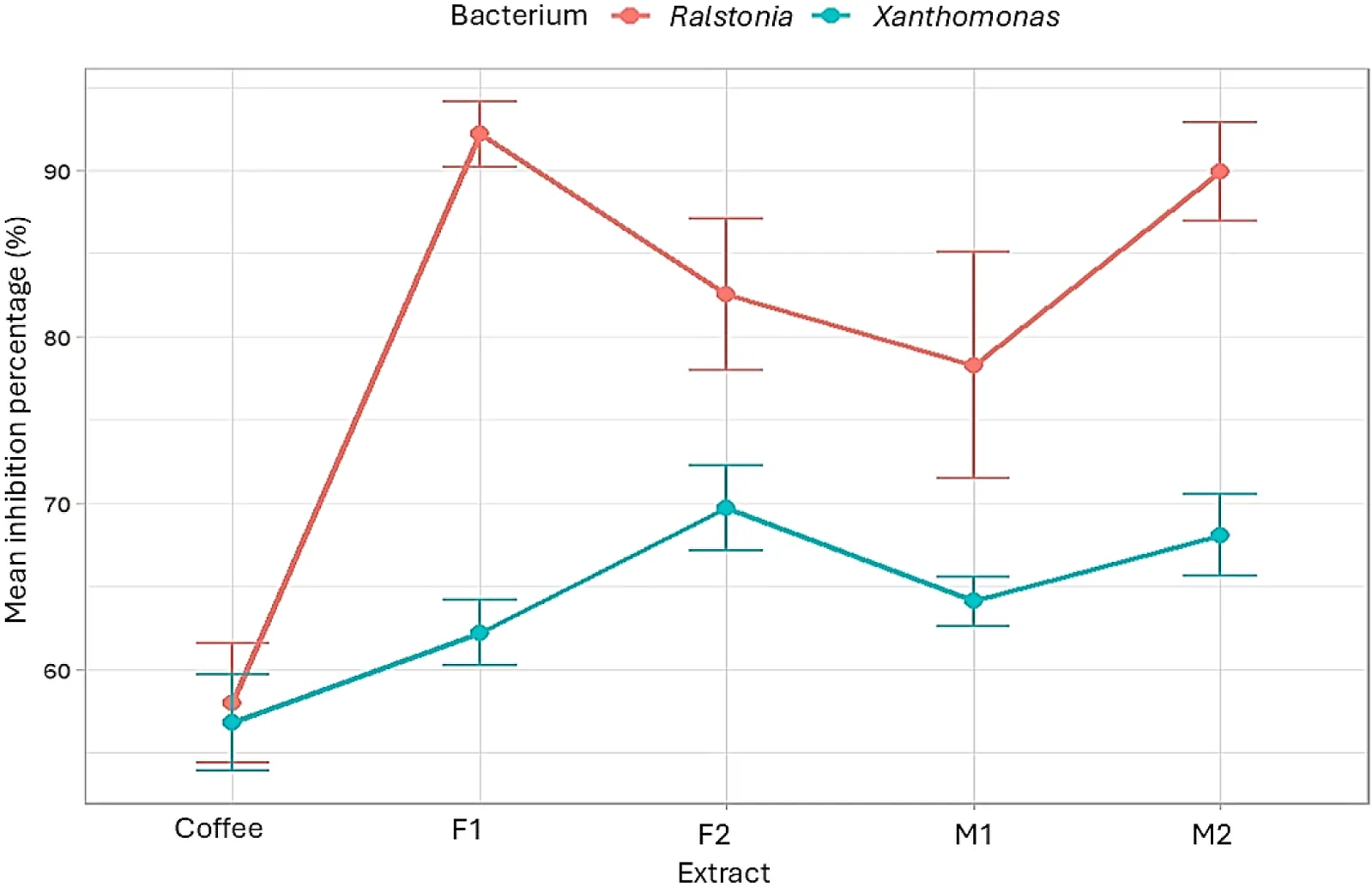
Inhibition percentage against the phytopathogenic bacteria studied using concentrated coffee, M1, and M2 extracts and fractions F1 and F2. *Ralstonia* is shown in pink, while *Xanthomonas* is shown in light blue.

Plant extracts are highly complex mixtures of chemical species with considerable inhibitory, antioxidant, and medicinal potential.^63^ Ultrahigh-performance liquid chromatography coupled with high-resolution mass spectrometry (UHPLC-HRMS) analysis of the concentrated coffee extract, M1, M2, and fraction F2 revealed the presence of a shared phytochemical profile (Table S6). This profile included common polyphenols such as gallic acid, catechin, and caffeoylquinic acids, as well as alkaloids (theobromine and xanthine) and polyols (mannitol and sorbitol), which may be associated with metabolic pathways intrinsic to the plant.^64^

When the extracts were analyzed individually, the coffee extract exhibited a wide diversity of bioactive compounds associated with different metabolic pathways including phenylpropanoids, flavonoids, terpenes, lipids, and polyketides. This composition is consistent with previous reports on coffee-pulp extracts, which was expected since this extract was not subjected to hydrothermal treatment.^8,65–67^

In contrast, the extracts subjected to elevated temperature and pressure showed the absence of quercetin and rutin, compounds widely recognized for their high antioxidant capacity,^67^ likely as a consequence of thermal degradation. However, these extracts contained other compounds of interest, including catechin and lignans, in M1. Catechin has been reported to affect bacterial cells at the membrane level by inducing oxidative stress and disrupting membrane integrity.^68^ Similarly, lignans exert antimicrobial activity through membrane permeabilization, membrane damage, and depolarization, thereby affecting cellular pH homeostasis and ionic gradients.^69^

M2 displayed a phytochemical composition similar to that of M1; however, indole derivatives (such as indole-3-ethanol) and aromatic phenolic aldehydes (including vanillin and syringaldehyde) were additionally detected, likely as a result of a greater extent of lignin degradation. Substituted indole derivatives exhibit a broad spectrum of biological activities, including antimicrobial effects, and have therefore been widely used as precursors for the synthesis of alkaloids and agrochemicals.^70^ Vanillin has demonstrated inhibitory effects against pathogenic bacteria through membrane pore formation, disruption of cellular homeostasis, and inhibition of energy metabolism.^71^ Likewise, syringaldehyde has been reported to possess antimicrobial, antioxidant, anticancer, and anti-inflammatory properties.^72^ These characteristics suggest that in addition to sharing the phytochemical profile of the coffee extract, M2 contains aromatic degradation products that may contribute to its inhibitory activity.

Furthermore, oxylipins were detected in both M2 and fraction F2. These compounds originate from the oxidation and subsequent conversion of polyunsaturated fatty acids and are known to exhibit broad antimicrobial activity, often displaying pathogen-dependent specificity. Due to their lipid-derived nature, their mode of action is believed to involve interactions with the cellular membrane.^73^

It is noteworthy that although F2 contained phenolic compounds such as gallic acid and oxylipins, it differed from M1 and M2 by exhibiting a more pronounced lipidic and peptidic profile. This characteristic is attributed to the presence of lipids and amphiphilic compounds, including 10-hydroxydecanoic acid and lauric acid, both of which are recognized antimicrobial lipids. These single-chain lipid amphiphiles destabilize bacterial cell membranes and contribute to antimicrobial activity.^74^

UHPLC-HRMS analysis demonstrated that the coffee extract possessed a complex polyphenolic profile characterized by the presence of glycosylated flavonoids and triterpenes. In contrast, the hydrothermally treated extracts exhibited a broader range of chemical groups including phenolic aldehydes, indoles, oxylipins, and amphiphilic lipids. Owing to their chemical nature and reported mechanisms of action, these compounds may explain the enhanced inhibitory activity observed in the hydrothermally treated extracts.

## Conclusions

The total polyphenol content of coffee pulp (220 ± 9 mg GAE/g DW) makes it an important source of these bioactive compounds. In addition, its composition, rich in cellulose (21.4%), hemicellulose (37.6%), and lignin (16.6%), positions it as a valuable residue with potential use in various areas.

Ultrasound-assisted extraction proved to be the most effective technique compared with high-power sonication for polyphenol extraction. However, the main effects analysis showed that the factor with the greatest influence on extraction was the biomass ratio, concluding that the best conditions for the ultrasonic bath were 40 min, 40 °C, and a ratio of 1 g:12 mL. Under these conditions, polyphenol contents of 220 ± 9 mg GAE/g DW for coffee, 220 ± 8 mg GAE/g DW for M1, and 228 ± 5 mg GAE/g DW for M2 were obtained.

The indicator bacterium *Micrococcus luteus* did not exhibit inhibition when the initial extracts were tested. Therefore, the extracts were concentrated by modifying the biomass-to-solvent ratio during the extraction step to 1 g:3.6 mL. This adjustment increased the total polyphenol content to 1,100 ± 200 mg GAE g^–1^ DW for the coffee extract, 2,100 ± 300 mg GAE g^–1^ DW for M1, and 1,300 ± 100 mg GAE g^–1^ DW for M2, resulting in inhibitory activity against both the indicator bacterium and the phytopathogenic bacteria.

Inhibition percentages against *Xanthomonas* were 57 ± 7%, 64 ± 4%, and 68 ± 5% for the coffee extract, M1, and M2, respectively. Similarly, inhibition percentages against *Ralstonia* were 58 ± 10%, 82 ± 17%, and 90 ± 7% for the same extracts. Following fractionation of the M2 extract using cellulose acetate filters, fraction F1 exhibited inhibition percentages of 62 ± 4% against *Xanthomonas* and 92 ± 4% against *Ralstonia*, whereas fraction F2 showed inhibition percentages of 70 ± 6% and 82 ± 10% against *Xanthomonas* and *Ralstonia*, respectively.

UHPLC-HRMS analysis revealed that the concentrated coffee extract possessed a complex polyphenolic profile characterized by a predominance of glycosylated flavonoids and triterpenes. In contrast, concentrated extracts obtained from the hydrothermally treated solids contained distinct chemical groups including phenolic aldehydes, indoles, oxylipins, and amphiphilic lipids. These compounds are likely responsible for the enhanced inhibitory activity observed in the hydrothermal treatment of the extract.

## Supplementary Material



## Notes

Algorithm for the inhibition test, comparation chart among ultrasound-assisted technologies, statistical support for 2^3^ factorial design, and representations of the inhibition zones in *Ralstonia* and *Xanthomonas* bacteria (PDF)

## Abbreviations

M1: extract obtained by solid residue for hydrothermal treatment at conditions 1
M2: extract obtained by solid residue for hydrothermal treatment at conditions 2
F1: fraction of M2 using a 0.40 μm cellulose acetate microfilter
F2: fraction of M2 using a 0.20 μm cellulose acetate microfilter
