# Supplementary material for: Hydrothermal Treatment Modifies the Chemical Profile of Coffee Pulp (Coffea arabica) Extracts and Improves Their Antibacterial Activity against Xanthomonas and Ralstonia
Source: ACS Omega. 2026 Oct 23;11(43):67227–37. doi: 10.1021/acsomega.6c07937 (PMC13737993; doi:10.1021/acsomega.6c07937)
Supplement: Supplementary file 1 [file ao6c07937_si_001.pdf]

Hydrothermal treatment modifies the chemical profile of coffee pulp (*Coffea arabica*) extracts and improves their antibacterial activity against *Xanthomonas* and *Ralstonia*

Eddy Jirón-García[1], Karina Rodríguez-Mora[2], Lidieth Uribe-Lorío[3], Cesar Bernal-Samaniego[1]*

[1]Sede Regional del Caribe, Universidad de Costa Rica, Limón, Limón 70101.

[2]Instituto de Investigaciones en Ingeniería (INII), Universidad de Costa Rica, San Pedro, San José 2060,

[3]Centro de Investigaciones Agronómicas, Universidad de Costa Rica, San Pedro, San José 2060

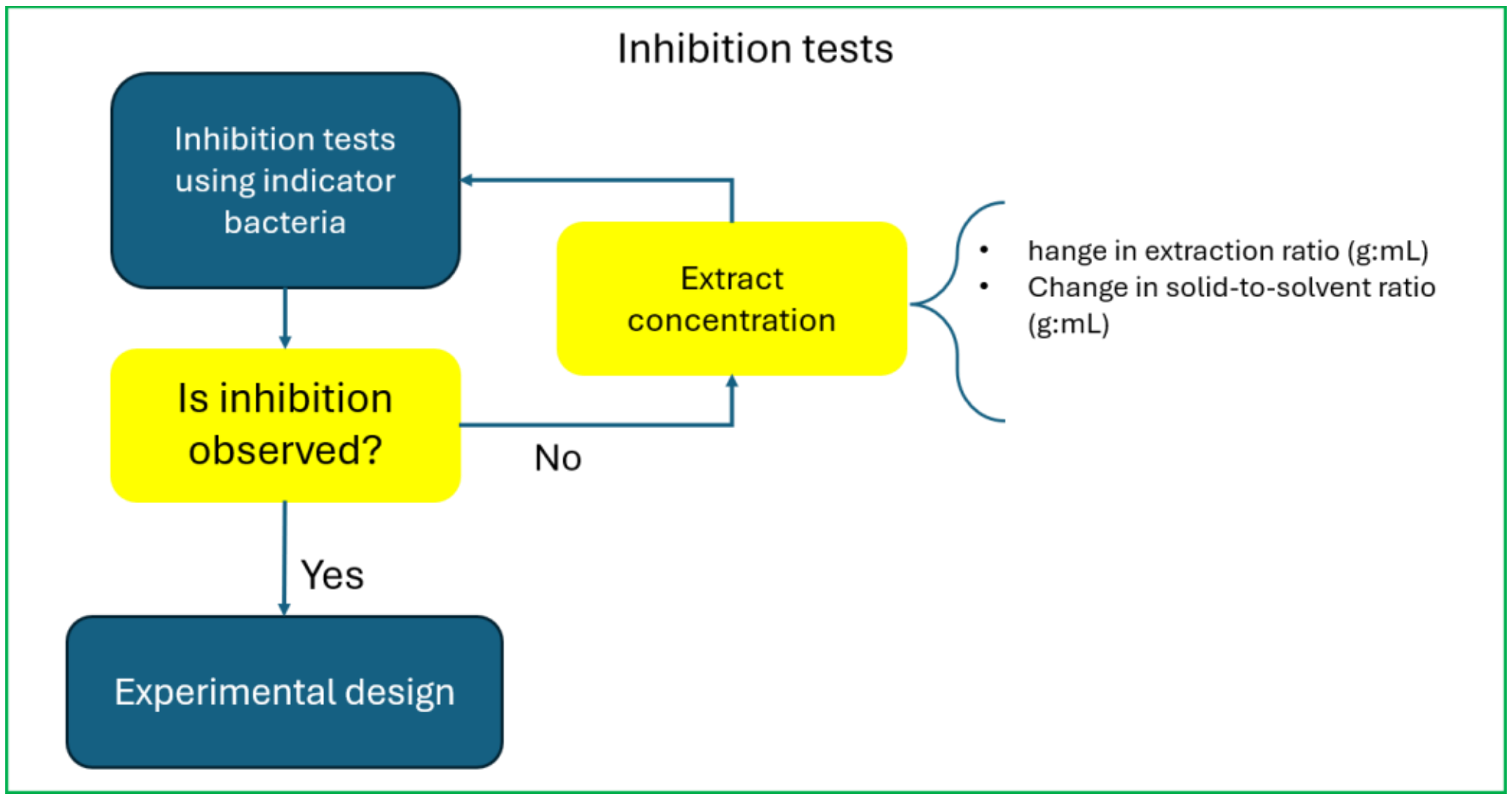

Figure S1. Experimental workflow for the preliminary inhibition screening and concentration of the extracts

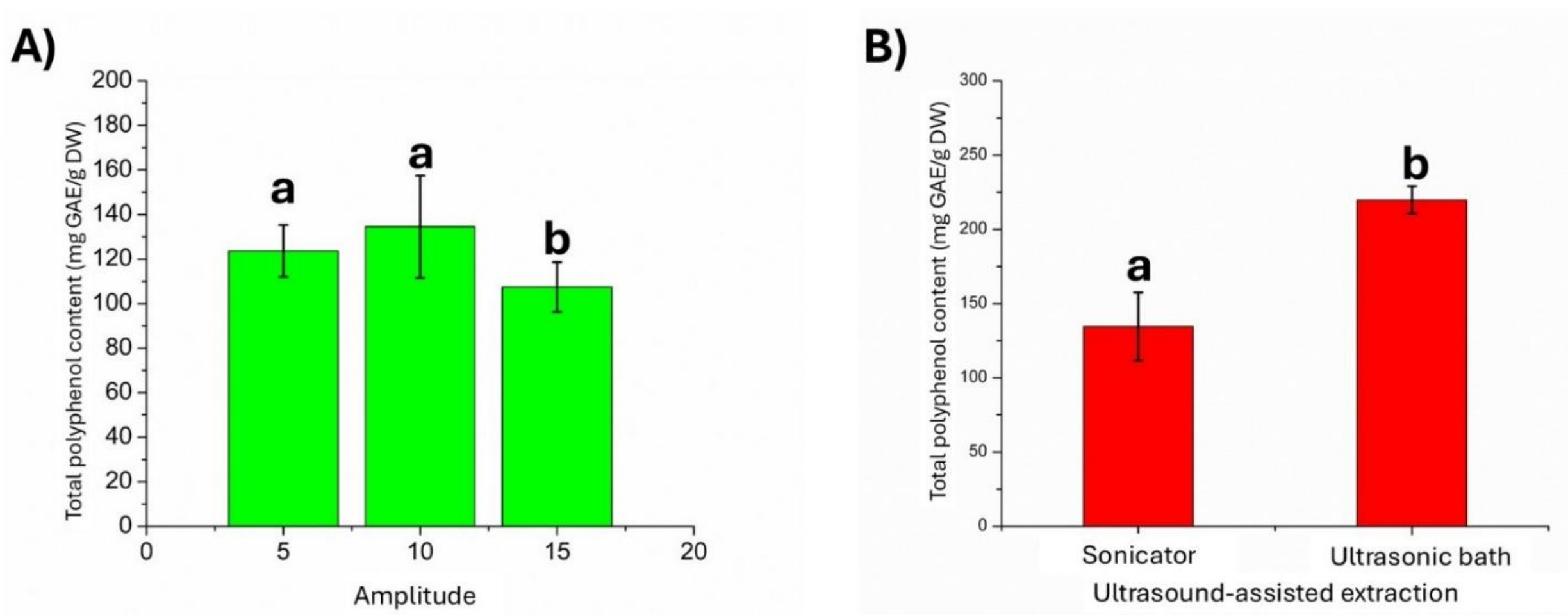

Figure S2. Total polyphenol content using A) extraction with a high-power sonicator using 50% methanol as solvent and a 1 g:25 mL ratio, and B) comparison between ultrasound-assisted extraction approaches using the mean value obtained with the high-power sonicator (Sonicator) at an amplitude of 10 and the overall mean of the measurements obtained using the ultrasonic bath (Ultrasonic). Bars represent the standard deviation; a and b indicate differences between treatments.

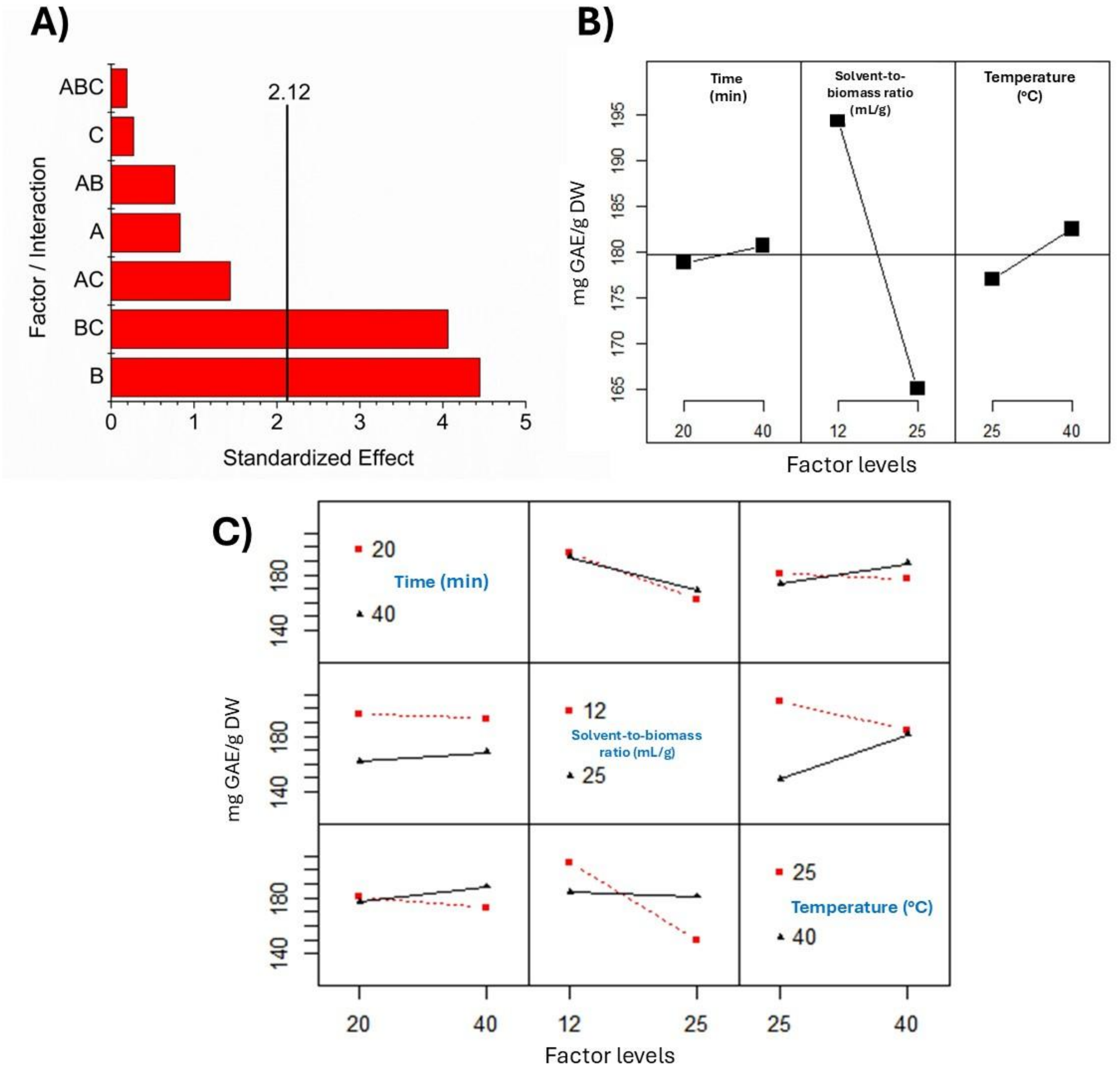

Figure S3. Results of the 2^3 factorial statistical analysis of the ultrasonic bath, where A) shows the Pareto chart of standardized effects, B) shows the main effects plot, and C) shows the interaction plot.

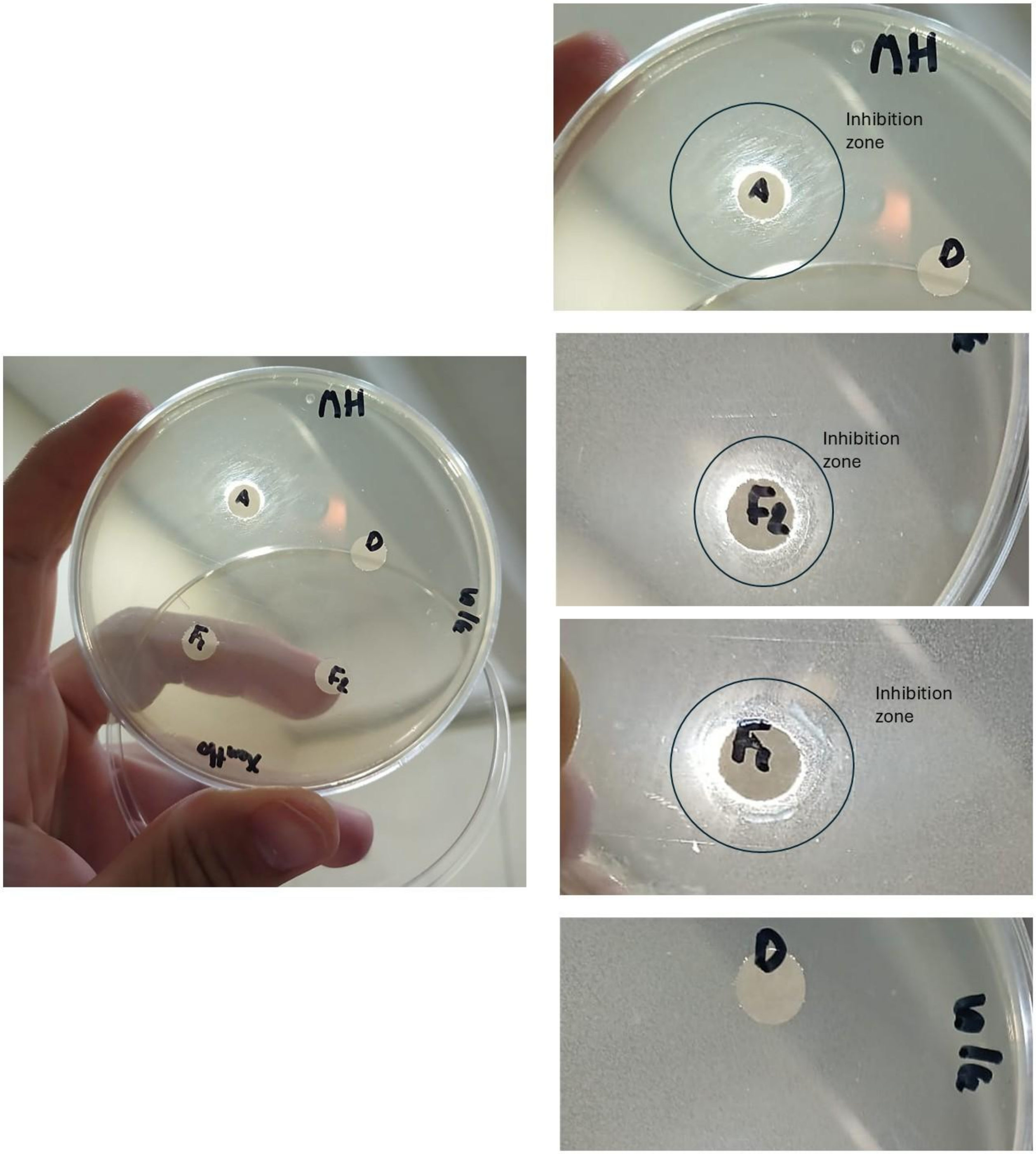

Figure S4. Representation of the inhibition zones in *Xanthomonas* using the F1 and F2 extracts, together with gentamicin at 0.1 mg/mL as the positive control (A) and 50% methanol as the negative control (D).

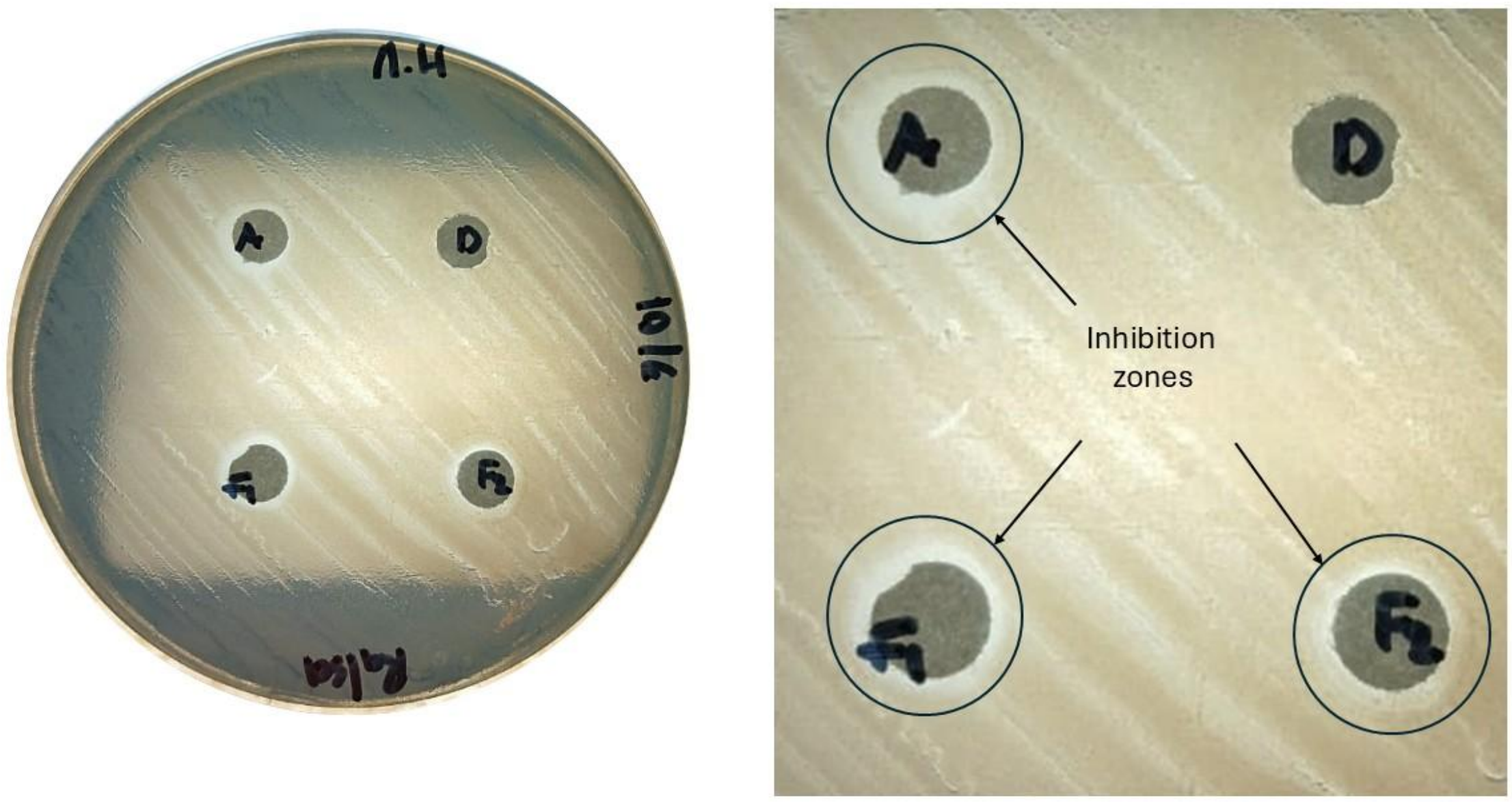

Figure S5. Representation of the inhibition zones in *Ralstonia* using the F1 and F2 extracts, together with gentamicin at 0.1 mg/mL as the positive control (A) and 50% methanol as the negative control (D).

Table S1. Experimental data from the disk-diffusion antibacterial assay of the untreated coffee-pulp extract against *Xanthomonas* and *Ralstonia*.

Bacterium	Extract	Replicate	Negative-control inhibition-zone diameter (cm)	Positive-control inhibition-zone diameter (cm)	Extract inhibition-zone diameter (cm)	Inhibition (%)
Xanthomonas	Coffee	1	0.0	1.9	1.0	53
Xanthomonas	Coffee	2	0.0	1.9	0.9	47
Xanthomonas	Coffee	3	0.0	1.9	1.0	53
Xanthomonas	Coffee	4	0.0	1.7	1.1	65
Xanthomonas	Coffee	5	0.0	1.7	1.0	59
Xanthomonas	Coffee	6	0.0	1.7	1.1	65
Ralstonia	Coffee	1	0.0	1.8	0.8	44
Ralstonia	Coffee	2	0.0	1.2	0.7	58
Ralstonia	Coffee	3	0.0	1.1	0.7	64
Ralstonia	Coffee	4	0.0	1.2	0.8	67
Ralstonia	Coffee	5	0.0	1.6	0.7	44
Ralstonia	Coffee	6	0.0	1.1	0.8	73
Ralstonia	Coffee	7	0.0	1.4	0.8	57
Ralstonia	Coffee	8	0.0	1.4	0.8	57

Table S2. Experimental data from the disk-diffusion antibacterial assay of the M1 extract obtained from the solid residue after 1 h of hydrothermal treatment against *Xanthomonas* and *Ralstonia*.

Bacterium	Extract	Replicate	Negative-control inhibition-zone diameter (cm)	Positive-control inhibition-zone diameter (cm)	Extract inhibition-zone diameter (cm)	Inhibition (%)
Xanthomonas	M1	1	0.0	1.6	1.1	69
Xanthomonas	M1	2	0.0	1.6	1.0	63
Xanthomonas	M1	3	0.0	1.6	1.1	69
Xanthomonas	M1	4	0.0	1.6	1.0	63
Xanthomonas	M1	5	0.0	1.8	1.1	61
Xanthomonas	M1	6	0.0	1.8	1.1	61
Ralstonia	M1	1	0.0	1.1	0.9	82
Ralstonia	M1	2	0.0	1.1	0.9	82
Ralstonia	M1	3	0.0	1.0	1.0	100
Ralstonia	M1	4	0.0	1.5	1.0	67

Table S3. Experimental data from the disk-diffusion antibacterial assay of the M2 extract obtained from the solid residue after 3 h of hydrothermal treatment against *Xanthomonas* and *Ralstonia*.

Bacterium	Extract	Replicate	Negative-control inhibition-zone diameter (cm)	Positive-control inhibition-zone diameter (cm)	Extract inhibition-zone diameter (cm)	Inhibition (%)
Xanthomonas	M2	1	0.0	1.5	1.1	73
Xanthomonas	M2	2	0.0	1.5	1.1	73
Xanthomonas	M2	3	0.0	1.6	1.0	63
Xanthomonas	M2	4	0.0	1.6	1.1	69
Xanthomonas	M2	5	0.0	1.6	1.0	63
Ralstonia	M2	1	0.0	1.1	1.0	91
Ralstonia	M2	2	0.0	1.3	1.1	85
Ralstonia	M2	3	0.0	1.1	1.0	91
Ralstonia	M2	4	0.0	1.2	1.2	100
Ralstonia	M2	5	0.0	1.2	1.0	83

Table S4. Experimental data from the disk-diffusion antibacterial assay of the F1 fraction obtained from the M2 extract against *Xanthomonas* and *Ralstonia*.

Bacterium	Extract	Replicate	Negative-control inhibition-zone diameter (cm)	Positive-control inhibition-zone diameter (cm)	Extract inhibition-zone diameter (cm)	Inhibition (%)
Xanthomonas	F1	1	0.0	1.6	0.9	56
Xanthomonas	F1	2	0.0	1.6	1.0	63
Xanthomonas	F1	3	0.0	1.6	1.1	69
Xanthomonas	F1	4	0.0	1.8	1.1	61
Xanthomonas	F1	5	0.0	1.6	1.0	63
Ralstonia	F1	1	0.0	1.1	1.1	100
Ralstonia	F1	2	0.0	1.0	0.9	90
Ralstonia	F1	3	0.0	1.0	0.9	90
Ralstonia	F1	4	0.0	1.0	0.9	90
Ralstonia	F1	5	0.0	1.1	1.0	91

Table S5. Experimental data from the disk-diffusion antibacterial assay of the F2 fraction obtained from the M2 extract against *Xanthomonas* and *Ralstonia*.

Bacterium	Extract	Replicate	Negative-control inhibition-zone diameter (cm)	Positive-control inhibition-zone diameter (cm)	Extract inhibition-zone diameter (cm)	Inhibition (%)
Xanthomonas	F2	1	0.0	1.6	1.2	75
Xanthomonas	F2	2	0.0	1.6	1.1	69
Xanthomonas	F2	3	0.0	1.6	1.2	75
Xanthomonas	F2	4	0.0	1.8	1.1	61
Xanthomonas	F2	5	0.0	1.6	1.1	69
Ralstonia	F2	1	0.0	1.1	1.1	100
Ralstonia	F2	2	0.0	1.0	0.8	80
Ralstonia	F2	3	0.0	1.0	0.8	80
Ralstonia	F2	4	0.0	1.0	0.8	80
Ralstonia	F2	5	0	1.1	0.8	73

Table S6. Comparative summary of compounds annotated by HPLC-HRMS in the coffee pulp extract, M1, M2, and F2

Chemical group	Putatively annotated compound	Sample(s) in which the annotation was reported	Ionization mode	Reported m/z	Annotation platform
Phenolic acids	Gallic acid/gallate	Coffee extract, M1, M2, F2	Negative	169.014	GNPS; SIRIUS
Flavanols	Catechin	M1	Negative	289.072	SIRIUS
Caffeoylquinic acids	Caffeoylquinic acid isomers	Coffee extract, M1	Negative	353.088	GNPS; SIRIUS
Caffeoylquinic acids	Dicaffeoylquinic acid isomers	Coffee extract	Negative	515.120	GNPS
Caffeoylquinic acids	Chlorogenic acid	Coffee extract	Positive	355.102	GNPS
Flavonols	Quercetin	Coffee extract	Negative	301.035	GNPS
Flavonols	Rutin	Coffee extract	Positive and negative	611.161; 609.146	GNPS
Flavonols	Kaempferol glycosides	Coffee extract	Positive and negative	595.166; 447.094	GNPS
Flavanones	Naringenin	Coffee extract	Negative	271.061	GNPS
Flavanones	Pinocembrin	Coffee extract	Negative	255.066	GNPS
Triterpenes	Tomentosic acid	Coffee extract	Negative	503.338	SIRIUS
Triterpenes	Tomentic acid	Coffee extract	Negative	487.343	SIRIUS

Alkaloids	Theobromine	Coffee extract, M1, M2, F2	Positive	181.072–181.073	GNPS; SIRIUS
Alkaloids	Xanthine/p-xanthine	Coffee extract, M1, M2, F2	Positive and negative	181.072; 179.058	SIRIUS
Polyols	Mannitol	Coffee extract, M1, M2, F2	Positive	165.076; 183.087	GNPS
Polyols	Sorbitol	M1, M2, F2	Positive and negative	183.086; 163.061	SIRIUS
Lignans	Syringaresinol	M2, F2	Positive	401.160	SIRIUS
Indole derivatives	Indole-3-ethanol	M2	Positive	144.081	SIRIUS
Phenolic aldehydes	Vanillin	M2	Positive	153.060	GNPS
Phenolic aldehydes	Syringaldehyde	M2	Positive	183.065	GNPS
Oxylipins	Trihydroxyoctadecadienoic acid derivatives	Coffee extract, M2, F2	Negative	327.218; 655.443	GNPS; SIRIUS
Hydroxy fatty acids	10-Hydroxydecanoic acid[1]	F2	Negative	169.124	SIRIUS
Fatty acid derivatives	Lauryl diethanolamide	Coffee extract, M1, M2, F2	Positive	288.253	GNPS
Cyclic peptides	Cyclo(Leu-Phe)	M1, M2, F2	Positive	261.159	GNPS
Cyclic peptides	Cyclo(Leu-Leu)	M2, F2	Positive	227.174	GNPS
Dipeptides	His-Pro	M1, M2, F2	Positive	235.119	GNPS
Dipeptides	Phe-Pro	M1, M2, F2	Positive	245.129	GNPS

Note: The table summarizes the annotations used for the comparative description of the chemical profiles in the manuscript. Differences in the reported *m/z* for the same compound reflect the ion or adduct detected under the corresponding analytical conditions.
